# Moral Physiology; or, the Priest and the Physician

**Published:** 1848-10-01

**Authors:** 


Art. VI.—*Essai sur la Théologie Morale, considerée dans ses rapports avec la Physiologie et la Médicine; Ouvrage spécialement destiné au Clergé.* Par P. J. C. Debreyne, Docteur en Médicine de la Faculté de Paris, &c. Quatrième Edition. Paris et Lyon.

*Essay on Moral Theology, considered in relation to Physiology and Medicine; designed especially for the Clergy.* By P. J. C. Debreyne, Doctor of Medicine, &c. Fourth Edition. Paris.

The intimate connexion between the sciences of medicine and theology is not sufficiently appreciated. The priest should be, at the present day, what he ever was, the *lux mundi,* the torch of knowledge, the treasury of the arts and sciences. In fact, he should be the man of the future, the precursor of intellectual, moral, and social progress. In this way he may regain the power he exercised formerly in society. That he should keep pace with, and even forerun his time, in scientific knowledge, is a truth which should be impressed on every clergyman. It is a social necessity of signal importance. But all branches of knowledge are not equally desirable.

Theology and medicine have ever been regarded as sister sciences. They seem to have a natural affinity to each other. Both are founded on charity. The Priest and the Physician have, from time immemorial, been the same. Often have the two professions been united in the same person. In times of idolatry, the heathen priest was skilled in medicine. The temples of false gods were thronged with sick, as suppliants for the relief of bodily infirmities. Freely and sincerely was it given. It could not be otherwise. Genuine piety ever prompts the heart to feelings of benevolence and acts of mercy.

Christianity is the fountain of charity. Its doctrines inculcate the practice of every social virtue, and the life of its founder was one perpetual illustration of the beauty of the theme. He went about doing good. Christ himself was styled the Great Physician. The Saviour of the world was always healing the sick. Profiting by this example, the Christian priesthood have attempted the alleviation of bodily infirmities while administering spiritual consolation. This practice, in its literal sense, is no longer needed. The motive spirit only is required. No man is fit to teach Christ's doctrines, who is not prepared to imitate his conduct towards the afflicted. He whose soul has not been melted to pity is little adapted to advance the interests of the Saviour upon earth, for *Deus charitas est.*

The medical man, on the contrary, is often called upon to be the priest. At the bedside of his dying patient, when the spirits are humbled by suffering, and nature is exhausted by disease, he is often able to drop the honey-dew of comfort, and smoothe the pillow of despair. Gratitude aids him in his task. When recruited through his agency, he is able, by a friendly word, to check the storm of passion, and divert the mind into a virtuous channel. Thus we may hope that, through the pious physician's means, many an errant soul has been redeemed. In truth, the two offices are inseparable to a certain extent. The physician is the priest, and the priest is the physician.

An intimate union, therefore, existing between divinity and physic, we come now to consider the kind and species of knowledge, in the department of medical science, likely to be most serviceable to the clergyman. We by no means wish him to have a regular medical education. All that is required is, that he should have an acquaintance with the general principles of physiology, and more especially of those parts which tend to elucidate the workings of the human mind and heart, and the influence of constitution upon character and manners.

There is an ever-prevailing tendency to carry things to extremes. An old writer compared the course of man to that of a chariot on a wide but dangerous road. Urged onwards by the passions, the feeble hand of Reason tries in vain to restrain the fiery coursers. Destruction threatening on the one side, she snatches at the reins, and rushes into equal danger in the opposite direction. Thus, by a series of gyrations is the course maintained—a steady, undeviating transit never is attained. In avoiding Scylla he runs into Charybdis. The *modus in rebus* is disregarded, and he loses sight altogether that there are—

> "Certi denique fines,
> Quos ultra citraque nequit consistere rectum."

The ars medendi is no exception to this rule. In former times, medicine was both styled and treated as a mystery. None but the initiated were admitted within the sacred precincts of the temple of Æsculapius. The prying eye of vulgar curiosity was carefully excluded by a veil of talismanic symbols. Evils, and those not few or trifling, were doubtless attendant on this system, and it was a cloak to much quackery and imposture. In recent times all this is changed. There is no longer mystery in medicine. The book of nature is laid open, so that all who choose may read. It is doubtful, though, whether this is not carried too far. In steering clear of charlatanism, the dignity of the profession has, we think, been lowered, by exposing its weaknesses to the vulgar gaze. The audience part of the public has been admitted too much behind the scenes; has been let too much into the secrets of the performance. We maintain that, in order to its full success, the medical, like every other, character, requires to be somewhat sustained by adventitious circumstances. Inasmuch as its influence, allowedly, depends upon imagination, and the confidence and hope inspired by superior attributes, the mask should not too hastily be torn aside, and the actor revealed with all his human frailties and infirmities.

When we consider, however, the great advancement of science occasioned by free discussion, we should be little inclined to revert to the close-borough system of our forefathers. The middle course is here decidedly the best.

A moderate but sound knowledge of the main principles of medicine would often be most useful to those who, by their talents, station, or official position, necessarily exert influence over their fellow men. This influence may be for good or for evil, according to their knowledge. They are constantly consulted by their more humble neighbours, and their advice is implicitly—nay, blindly followed.

To the members of the clerical profession do these remarks, with great propriety, apply. By their engagements thrown into the bosom of society, they are generally the repositories of all its sorrows. The inmost secrets of the soul and body are alike revealed to them, and their counsel is sought in the utmost depths of misery and degradation. Often by a timely word may the friendly pastor convey comfort to a heart breaking under the anguish of shame, and hope to one throbbing with despair. But in order to apply the balm effectually, he should be intimately acquainted with the nature of the wound. To administer advice and consolation, he should be aware of the failings of his patient, and how much is due to temperament and constitution. In fact, the good priest should have a tolerable acquaintance with physiology, in order to be most useful in his vocation. For knowledge, in this instance, most certainly is power—the power of doing good. The foundation of the priestly influence should, doubtless, be laid in the exercise of active benevolence—in that charity which is at once the bond of society and basis of civilization.

It will be perceived from the above, that we make no allusion to even the most elementary details of *practical* medicine. These should be avoided, as we consider all popular medicine, however simple it may be, more injurious than useful. The reason is, that people of the world, whether literati or priests, apply their remedies at improper times or in ill-regulated doses. And even if they employ but simple, inoffensive, medicaments, their assistance, generally more assiduous than enlightened, is prejudicial, inasmuch as it leads to a loss of valuable time. The opportunity of arresting the progress of the malady is lost, and the disease, hence, becomes incurable or mortal.

M. Debreyne, in the work before us, has treated some of the points most serviceable for the priest. The book is not a regular, even an elementary, treatise on physiology, but a series of essays on physiological and practical subjects, without any very definite order and arrangement. On the whole, he has discussed them with learning and ability, and shown their bearings on moral theology. Written expressly for the benefit of the Catholic clergy, to aid them at the confessional, some topics are discussed which we think had better have been avoided, as we question whether the priest should ever be consulted on such matters. We shall pass them by altogether, and select others more calculated to interest our readers.

Of all methods for ascertaining, by the organization, the amount of human intelligence, M. Debreyne prefers that of Camper. He calls it the *phreno-metric method*. He is decidedly opposed to phrenology, and sums up his objections in these words:—

"Phrenology has never appeared to me worthy of a serious discussion. As a psychological system, it is a contradictory conception. As an anatomico-physiological theory, it is an hypothesis completely gratuitous. It is expressly worthy of notice, that none of the French zoologists of this age, who have so profoundly studied the organization of life and the principles of physiology, have paid attention to it. Cuvier never mentioned it without disdain. MM. Blainville, Geoffrey Saint Hilaire, Serre, Flourens, Dutrochet, Duméril—in fact, all the physiologists who have an European reputation, have passed it over. It is the same in England. With the exception of Mr. Combes, who is a man of talent, and moreover the official champion of phrenology in England, as M. Broussais is in France, you can find no one to notice it. Throughout Germany, the cradle of organology, the name alone of this pretended science is acknowledged."

Comment upon this were useless.

The doctrine of *temperaments* is now universally recognised in the science of medicine. The influence of a predominance of one system of organs over the rest is well established. Again, it were waste of time, at the present day, to seek to demonstrate the amazing influence of the physical over the moral. This truth has been so often repeated by persons even of the most mediocre talent, that it has become quite common-place. But there is one circumstance which is not so much heeded. We mean the immense power exercised by temperament upon the moral and social faculties of man. This influence is felt not only on his intellect and genius, but upon his character, his humour, tastes, and inclinations, and even upon his morality and sociability, his happy disposition to virtue, or his unfortunate propensity to vice; in fact, upon the destination of the soul itself. This of course is dangerous ground and must be trod with care, as it almost implies a doubt of man's responsibility; but it is a subject of too much interest to be passed over in silence.

To the usual list of pure or primitive temperaments, M. Debreyne has added one which he calls the *erotic*. This term will readily be recognised, but whether the erotic disposition is so distinctly marked as to deserve the rank of a distinct temperament, any more than a dozen other strong propensities, all doubtless dependent upon organization, may be fairly questioned. However, the notion, as far as we know, is original.

The physical attributes and historical illustrations of the various pure and mixed temperaments are of course familiar to our readers. The moral attributes are, we presume, less so, and therefore we need not apologise for their introduction. In the opinion of our author, the following are the intellectual, moral, and social faculties of a man of *sanguine* temperament. These are his characteristics, tastes, passions, vices, and virtues. He says—

"With the sanguineous, the sensations are most lively; the intellectual functions are exercised with ease and freedom; the memory is good, the imagination lively and brilliant. Apprehension is quick. They readily comprehend that which is taught them, but they pass rapidly from one idea to another, show little solidity in their conceptions and ideas, are unsuited to profound or close meditation, and sciences of observation, because they are too hasty in drawing conclusions and forming definite judgments. They are better fitted for works of imagination. With little reflection and ill-sustained attention, they pretend to reason upon everything. They are superficial upon all questions, but profound in none. You rarely find a sanguine man acquire erudition, for patience is required for scientific investigation. Rarely, also, does he become a distinguished man, because his intellectual powers, always of a mediocre character, are not formed for long and abstract meditations. They are incapable of the higher flights of philosophy.

"The moral character of the man of sanguine temperament is marked by liveliness, sweetness of temper, generosity, sincerity, benevolence, cordiality, and attachment. He is honest, obliging, polite, gentle, humane, pitiful, affectionate, courageous. His manner is frank and open, his style facile and gay. He is easy of access, and pleasant in converse. But still, though jovial and pleasant, he is generally trivial and inconsistent. You can even say that triviality and inconsistency are his principal attributes. The dominant predilections of such a man are towards sensual pleasures, principally fleshly lusts, the enjoyments of the table, shows, balls, games, feats of strength, the chase, parties of pleasure, excessive vanity and foppishness. Sometimes they take the turn of love of adventure, of war, or of glory, but seek above all things change in pleasure. The *dominant vices* of the sanguineous are intemperance and incontinence. Being the man of pleasure, and all idea of pleasure being centered in his own person, he is the born foe to penitence, austerity, and Christian mortification. The appetites of the sanguine are fierce and ungovernable. Ever ruled by the law of their organization, and almost irresistibly impelled by the fire of their temperament and its accompanying passions, they are incessantly recalled to their pleasures, and on the point of yielding to the vices which are ever the bitter fruit. In general, you can little rely upon their promises and protestations of abnegation and fidelity, at least until you have succeeded so far as to inspire them with admiration for the beauty of virtue, or have made them attend to hygienic practices, such as temperance, manual labour, and prolonged exercise of the body. At first you must bring into operation, with this sort of persons, the virtues most akin to their nature, their character, and their taste. For instance, acts of generosity, offices of charity, benevolence, and well-regulated alms-giving. By degrees you will be able to retrench the luxury and superfluity of the table. For this, indeed, nothing is more serviceable than to severely exercise the body by manual labour, by which means the excess of nutriment is quickly dissipated. In addition, we should endeavour to deaden voluptuous sensations, and to mortify the pride of the flesh, and to habituate the body to a simple, frugal diet, which will ever be grateful when seasoned by hunger and fatigue."

In this way our author proceeds to point out the tendencies to vice or virtue engendered by temperament, with the most judicious method of management. This subject appears to us especially deserving the attention of those who have charge of the insane. They are, with respect to their patients, very much in the position of the Catholic clergy with regard to their flocks. In both cases, they have the entire, almost uncontrolled charge of their clients. Their object is equally to bring them back from an erring path into sober, intellectual, and moral sanity. Their engines and powers are nearly alike. They have to work upon and educate the mind by moral and physical means. How necessary, then, for each in his sphere, to avail himself of every aid for the investigation of character and propensity. When a disease is once known it is half cured.

We do not intend to follow M. Debreyne through his delineations of the muscular, bilious, lymphatic, or nervous temperaments, and their various combinations, but shall proceed at once to notice what he has to say upon the erotic or genital temperament in both sexes. This subject has, to a certain extent, been handled in a masterly manner by Esquirol and others, under the title of the genital *sense*. Esquirol especially pointed out its distinction from nymphomania and satyriasis. In these lattter derangements, he says, the mischief lies in the reproductive organs, the irritation from which reacts upon the brain. In erotomania the love is in the head. In the one class of cases the patients are victims of a real physical disorder; in the other they are the sport of their imaginations. The one is lust, the other honest love. M. Debreyne does not agree with all this. He does not believe that the affections of the heart in erotomania are honest affections: he believes they are real passions, more or less unchaste. This may be true, but the reason assigned for this opinion is odd enough:—" Because," says he, " the affections which are honest and legitimate, and have a praiseworthy object, are quiet, calm, and pacific, never turn men's heads, or render people silly." This doctrine seems so contrary to the recorded observations of all ages and countries, that we can scarcely give our assent to it. We are afraid M. Debreyne himself has never felt the tender passion.

"The erotic temperament," says our author, "does not present any physical attributes which are peculiar, or evident at first sight, if we except the large size of the cerebellum noticed by Gall. Perhaps it may have more certain characters, deduced from those of the sanguineous and nervous temperaments. Thus, by adding to the sanguineo-nervous temperament, an exalted sensibility and great development of the sexual system, we have the proximate cause of the erotic temperament. It shows itself usually by the display of all the sensations and all the desires which seem to have physical enjoyment for their object, or at all events no other end than generation.

"Doubtless it is to the erotic temperament that an immense number of individuals, in whom a wrong education and a false direction to the ideas and affections have left the will enslaved and the soul subdued to the empire of the senses, owe those excesses and deplorable derangements of which they are too often the unhappy victims. But, independently of a faulty organization, there is a host of causes, both physical and moral, capable of developing this temperament. For example, an ill-regulated life, a succulent and stimulating diet, idleness, abuse of alcoholic liquors, the reading of novels and love romances, attendance at theatres and balls, concerts, parties, masquerades, and exciting conversation. This is quite obvious. Now what can be done to counterbalance this distressing propensity to vice, and that fatal law of the flesh which stifles the spirit by its overwhelming weight? The best treatment here is a judicious management deduced from moral and hygienic precepts. The first thing, doubtless, is to inspire the fear of God and the love of virtue. Then, of course, all those remote and proximate causes above mentioned must be carefully avoided. Temperance and sobriety of demeanour must be inculcated. Manual labour, corporeal exercise, an incessant mechanical employment, sometimes even the chase, anything, in fact, to occupy the mind and fatigue the body, have produced in certain cases the most astonishing effects. Diana, we are told, is the natural enemy of Venus. Violent exercise stifles erotic sentiments, in giving rise to sensations still more imperious—such as an insatiable hunger, with an irresistible propensity to physical repose."

Many interesting physiological questions arise out of the subject of the Cæsarian section. Sacred or theological embryology is much more studied, and is of much more importance in Catholic countries than in Protestant; because the Romish church believes in the union of the soul with the body at the moment of conception, and therefore enjoins the baptism of the fœtus at any epoch of gestation it may have been aborted. The forms of this baptism vary according to circumstances. Thus, if it give evident signs of life, and is of human form, it is absolutely baptized, without reserve. If its life only is doubtful, this condition is attached—*si tu vivis, ego te baptizo, &c.* If both life and form are equally doubtful, they say,—*Si tu es homo et vivis, &c.* But either with or without the conditional form, everything in the shape of an embryo is baptized, provided always it is not in a state of decomposition or manifest disorganization. In this country, on the contrary, every one is aware that the practice is, to baptize only those children who live after birth; and the period of viability is stated by physiologists as not earlier than the sixth or seventh month of gestation. Whatever opinion we may entertain as to the value of discussion on embryology in a theological sense, we cannot doubt that the investigation has led to some new and interesting views in physiology; just in the same way as the search for the philosopher's stone by the alchemists led to the most brilliant discoveries in chemistry. We must not be understood, however, as wishing in the slightest degree to throw discredit on researches undertaken on conscientious, religious grounds. The motive, in some measure, qualifies the act.

Among the subjects discussed, arising out of the general disquisition on sacred embryology, may be enumerated,—the animation of the embryo; the period at which the soul becomes united to the body; abortion; embryogeny; proofs of the survival of the fœtus after the death of the mother; the signs of real and apparent death; and the occasions when it is necessary to perform the Cæsarian section. All these are discussed in the volume before us, with more or less ingenuity and learning. The first and second of these topics are interesting:—

Plato, Asclepiades, Protagoras, and several of the stoics, maintained that the reasonable soul does not exist before birth. The infant, say they, has the soul infused into it at the moment it is born. It is plain that they have taken the breath, *spiritus*, the *pneuma* of the Greeks, for the human soul. Aristotle was the first to fix animation at the *fortieth* day for boys, and at the *eightieth* or *ninetieth* day for girls. St. Augustine, St. Thomas, and all theologians after them, adopted the opinion of Aristotle, which since prevailed in the schools until the middle of the seventeenth century. The Penitentiary of Rome follows the dogma of Aristotle, without either examining or confirming it.

An immense number of facts proves that the distinction between the sexes above alluded to has no shadow of foundation. We may add, that it is most absurd and ridiculous. Moreover, it is extremely probable, not to say certain, that the fœtus is animated much earlier than is generally imagined. Cangiamila says that he saw an embryo of sixteen days which showed very evident signs of life. He cites a number of similar examples.

Many authors will not admit of animation until the principal members of the fœtus are formed. Zacchias believes that it occurs at the instant of conception. Basile would not allow of any distinction being made between the animate and inanimate fœtus, because he was persuaded that the soul is created at conception. St. Gregory of Nyssa declares, that it is inconsistent with good sense to admit that a thing inanimate has power to move and increase; yet this may be observed in the infant, from the very commencement of gestation. It must therefore have life. St. Cæsar was of the same opinion. Florentini thinks that it is probable that the embryo is animated immediately after conception. Consequently he teaches that we ought, under pain of committing deadly sin, to baptize the germ or embryo, even when it is no larger than a barley-corn and shows no sign of life, provided it be not decomposed or evidently dead. The reason he assigns for this practice is, that he believes the embryo animated—that is to say, already endowed with a reasonable soul. Cangiamila tells us that many celebrated theologians and physicians hailed the work of Florentini with marked approbation.

The faculties of theology of Paris, Vienna, and Prague gave their assent to the doctrine. That of Paris declared that the doctrine of Florentini was a safe one—*indubitata doctrina;* that it was useful, in preventing abortion among those irreligious women who procure it without scruple, under the pretence that the germ is not yet animated. This doctrine received equally the adhesion of the rector of the University of Rheims, of the University of Salamanca, and that of the faculties of medicine of Vienna and of Prague. A thesis was even defended publicly in the latter city, to maintain the proposition, that at the moment of conception the germ has a reasonable soul. Cangiamila himself, the celebrated author of the Grand Sacred Embryology, also teaches, that it is probable that the germ of the fœtus is animated immediately after conception.

"We" says M. Debreyne, "agree with St. Basile and Zacchias in sentiment; that is to say, we believe that animation takes place at the very moment of conception, and these are our reasons:—If the life of man ceases as soon as soul and body separate, we may infer that it commences when the soul is united to the body, however little this may be, or whatever its rudimentary form. But immediately the ovum is fecundated, it begins to grow. It grows because it lives, and it lives because it is animated. Hence the germ or human ovum is animated at the very instant of conception. Moreover, material life being dependent immediately on the sensitive faculty of the soul, and the sensitive faculty of the soul not being separable from the intellectual faculty of the soul, it follows that the reasonable soul is united with the embryo at the very instant of conception.

"Do we not know that the soul remains united to the body even till the last sigh of suffering humanity, even when most of the organs are struck with paralysis and death? Is that light puff of air, the last feeble remainder of organic life which will be extinguished in a few minutes, is that a vitality much superior to that of a fecundated embryo? The latter at least is a formative, plastic and increasing life. And let not our reason be obscured by the diminutive and shapeless condition of anything that contains the slightest portion of animated matter. You see no organs in the amorphous germ of a hen's egg. Aid your sight by a microscope, and you will immediately discover all the lineaments of the organization. I repeat it, let not natural diminutiveness obscure your reason. The Almighty is equally great and infinite in small things as in large, or rather in the material order of the universe, nothing is great or little in the eyes of God. These relative qualities of greatness and littleness result from the weakness of human intellect; necessarily feeble here below, to bring us into relation with the material world, that we may appreciate its order and harmony."

One of the main and most reasonable objections urged against the too frequent practice of the Cæsarian section has been, that the mother may not be *actually* but only *apparently* dead. She may be in a state of trance, or suspended animation, when, of course, such an operation would most probably be fatal. Hence the practice in this country and France, never to perform it unless the mother has died suddenly, by an accident or some other obvious and decisive cause, and the fœtus is believed, by the term of gestation, to be viable. In Sicily however, the law ordains that all women without distinction, who die during pregnancy, whatever may be the epoch, shall be opened, in order to confer baptism on the fœtus, or the embryo. The only question then to determine, is whether the woman be really dead. We believe that science is not put to so severe a test on any occasion in this country, but still the signs or evidences of death are always important in a medico-legal point of view. If we were called upon suddenly to perform the Cæsarian section upon a woman far advanced in pregnancy, we should be glad of any lights to aid us in determining whether she was really or actually dead, or was merely in a trance. Authentic instances have occurred before now of this operation having been performed upon persons in a state of suspended animation, or even of their having been buried in that condition. Let us now analyze the subject, with the aid of our author.

Writers on medicine in all ages have indicated the signs of death. Those generally enumerated in former times were:—The absence of circulation, respiration, heat, and sensation; a cadaverous countenance, a leaden, livid hue of the surface; a yellow colour of the palms of the hands and soles of the feet; a putrefactive odour, with dull weight of the body, &c. To the same purpose were trials by the mirror and lighted taper; surgical or operative proofs,—such as incisions into the sole of the foot, punctures, cauterizations, burnings, &c. It was found that if all these circumstances were united, they would not suffice to establish the certainty of death in all cases. It became, therefore, necessary to discover other and more certain signs. Modern authors have proposed four, which they consider infallible:—to wit: 1st. Commencing putrefaction. 2nd. Cadaveric rigidity. 3rd. A flaccidity or faded appearance of the eyes, with a glairy pellicle covering them, and a cloudy appearance of the cornea. 4th. Loss of muscular contractility under the shock of a galvanic battery. Let us briefly examine the respective value of these signs.

Putrefaction, without doubt, is a certain sign of death, and is even generally regarded as its only positive indication. But it is one almost impossible to obtain, on account of its tardy manifestation (from three to six days), for purposes of embryology, because we cannot wait for it. It is, therefore, most commonly a purely theoretical sign, scarcely of any value in practice. Cadaveric rigidity is one of the most sure and characteristic signs of death. If the limbs are flexible, provided always cadaveric stiffness has not preceded, you may presume that life is not extinct. It is stated in the *Dictionnaire des Sciences Medicales* that a girl eight years of age, who had fled from her father's house, was found seven days afterwards in a wood, deprived of sense, motion, circulation, and respiration; but her joints were flexible, and they knew by this circumstance alone that her death was but apparent. The celebrated Louis regarded cadaveric rigidity as the constant result of death, and its most certain indication. He said, that having made, during many years, uninterrupted researches upon more than five hundred persons just dead, he always found that at the moment that the vital actions ceased the articulations began to stiffen, even before the temperature of the body was lessened. Orfila, dean of the Faculty of Medicine, considers cadaveric rigidity as a sign as much to be depended upon as putrefaction itself. Nysten has shown by experiment that the rigidity is constant, even in individuals who have died from putrid diseases. But it is essentially necessary to distinguish the cadaveric rigidity of which we have been speaking, from convulsive, spasmodic, or tetanic rigidity. These are the distinctions, according to Louis and Nysten:—Nervous rigidity always precedes death, whether this be apparent or real; whereas the exact opposite is the case in cadaveric rigidity—namely, it is manifested some time after dissolution. Further, when by force we have overcome the convulsive spasm, the member speedily resumes its former position. But when we overcome the rigidity of real death, the limbs remain in the same situation in which we choose to place them. If dissolution has actually occurred, any convulsive spasm that may have been present ceases in an hour or two, and is infallibly succeeded by true cadaveric rigidity. We are told that the lower jaw, if dropped, does not go back again to its proper position if death is real. If it is only apparent, and produced by a nervous or spasmodic condition, it rises against the upper jaw. This is a sign which, under certain circumstances, is of value to confirm or invalidate other symptoms.

The flaccidity and shrivelled appearance of the eyes, and the obscurity or want of transparency and brilliancy in the cornea, is of such a kind, that those who look into the eyes of a corpse do not see their image reflected there as in those of a living person. Louis thinks these signs infallible. Dr. Vigné has verified them upon more than two thousand subjects, at the hospital of Rouen. This sign, together with that of the glairy pellicle, although of great value, may yet be absent in some cases of sudden death; as, for instance, violent apoplexy, the rupture of an internal aneurism, asphyxia by carbonic acid gas, and some diseases of the eyes.

Now for the absence of muscular contractility under galvanic influence. This is the result of experiments made by Nysten upon forty subjects. Muscular contractility was manifested in all, under the influence of the voltaic pile, but in very various degrees. Ordinarily the excitability was not extinguished until from six to fifteen hours after death; once at the end of an hour and a half; and at another time, at twenty-seven hours after dissolution. These are, therefore, the extreme terms. If we are to judge from these results, until further experiments are instituted, we conclude that muscular contractility is displayed in all dead bodies under the influence of the pile of Volta. This, therefore, proves neither the life nor the death of the subject, since this phenomenon is manifested equally upon one really defunct and one only apparently dead. But if we cannot say that life exists as long as muscular contractility continues, we can be certain that life is gone for ever if galvanism will no longer induce muscular contraction. Thus there is no exception to this rule, that, if after having displayed a muscle upon one of the limbs—an arm for example—we apply a current of electricity to it and produce no contraction, no motion of the fibres, it is an indisputable proof that the muscular contractility, or irritability, is completely extinguished, and that consequently death is certain. We do not hesitate to state our conviction, that we regard the complete extinction of muscular contractility, or excitability, as proved by galvanism, as a more satisfactory sign of death than the incipient putrefaction. For, in fact, the putrefaction may be merely apparent, as the death itself may be unreal. It was doubtless this possible deceptive putrefaction that induced the illustrious Louis to declare, that bad odour and decomposition were not always certain evidences of death.

From all that has been stated it results that, according to the very best authorities, two *practical* signs, or two orders of practical signs, indicate with certainty the reality of death. These are cadaveric rigidity—so called because it is peculiar to the dead, and softness, flaccidity, sinking and wasting of the eyes, with obscurity, and want of transparency and brilliancy of the cornea. These two signs, cadaveric rigidity and cloudiness of the eyes united, being, in our opinion, certain characteristic signs of real death, it follows that the contrary signs, also united, are irrefutable evidences of suspended animation. One of these two contrary signs is the permanent flexibility of the members, not preceded by cadaveric rigidity. We call that cadaveric rigidity which constantly ensues *after* death. Hence, according to this doctrine, a rigidity which shows itself an hour or two after death, but which began before death, or at the moment of decease, is not true cadaveric rigidity, but a diseased, nervous, convulsive, or tetanic stiffness or spasm. *Frigorific* rigidity, that is to say, that which is the effect of asphyxia by freezing, is general, and occupies all parts of the body. Even the abdomen is stiff like other parts, which is not the case in nervous rigidity. The second opposite sign is the permanent physiological state of the eyes—namely, their firmness and consistency, and the vital transparency, lustre, and brilliancy of the cornea. If then, these two signs, to wit, the permanent flexibility of the limbs not preceded by cadaveric rigidity—and everybody knows that the flexibility which succeeds the death-stiffness is an indication of incipient putrefaction—and the permanent physiological condition of the eyes, are found united, we can be certain that death is only apparent.

If galvanization, as we have seen, is the most sure, nay the only infallible mode of testing the reality of death, we must still confess that, for purposes of embryology, the galvanic proof is, after all, more scientific than useful. On this account recourse is rarely had to it; and for this reason—muscular excitability is not extinguished soon enough in dead bodies; since cases have been noticed where it remained for twenty, or even twenty-seven, hours after death. Hence this proof proves nothing. In other words, it is only negative. It is the absence of contractility which enables us to conclude with certainty that life is absolutely extinct. In order to be a valuable method of determination as to the reality of death, especially previous to performing the Cæsarian section upon women who have died during pregnancy, the excitability of the muscles under galvanic influence should invariably cease at a certain time after death; for instance, an hour or two at the farthest, instead of twenty-four or twenty-seven hours. Nevertheless, when there is a well-founded doubt as to the reality of death, recourse can be had to the galvanic test, since we have cited a case where the muscular irritability was extinguished an hour and a half after dissolution.

Among the disconnected essays in the work before us, we find one intitled, "A New Theory of Homicidal Monomania and Suicide." As this may present some points of interest, we think we cannot do better than select it for analysis. Most of the remaining topics have been already treated in former numbers of the Journal.

At the present day, all medico-legal practitioners admit of homicidal monomania without delirium. This term may be defined as that peculiar state in man, when, without showing any derangement of intellect, he is impelled by an irresistible desire, forced by a blind instinct, to do an action which his reason reproves and condemns. Beset by ideas of theft, arson, murder, or suicide, which he tries in vain to efface, he feels all the horror of such promptings, and yet his will is overpowered. Without motive or interest he steals, burns, kills, or destroys himself. Such is a sketch of the mental malady described by Esquirol, Pinel, Marc, Gall, &c., and now usually termed moral insanity.

M. Debreyne does not coincide in this theory, or at least will not admit the definition so long as the monomania consists only in *idea*, and does not pass to the *act*. According to him, all monomania which proceeds to the consummation of an act, is a maniacal monomania, at least at the moment of the execution of the act. That is to say, that then a sudden alteration takes place; there is a greater or less disturbance of the reason. This is sufficiently powerful to destroy moral liberty, and so pervert the will as to render it purely physical and animal.

"We," says he, "admit, then, this impulsion, this violent propensity, but not that it is absolutely irresistible, for in our eyes it does not become irresistible until the precise moment when the intellectual perturbation occurs. We believe that the doctrine of a temporary insanity, of a sudden eclipse of the reason at the time of the act, is preferable and more moral than the hypothesis of medical.jurists, who assert that the monomania, whether homicidal, suicidal, or incendiary, can impel to the consummation of the act, without mania or intellectual disturbance. We repeat, that we cannot admit this theory or principle of monomania with irresistible desire and without delirium during the act, because it appears to us dangerous, inasmuch as it suspends the course of freewill, is destructive of the morality of human actions, and tends to favour impunity for crimes. For if the impulse is irresistible, and is unaccompanied by delirium during the act, what becomes then of free will? Besides, in our mind, the disturbance of the reason will always be more comprehensible and conformable to the common sense of mankind, than a perversion of the will, united with an affective lesion without delirium during the act, which no man, after all, can satisfactorily establish.

"We believe, moreover, that the sudden and momentary disturbance of the reason is the consequence or effect of an unfortunate propensity which has not been sufficiently combated; or results from neglect in avoiding the occasions which foster and give it development. These circumstances or generative causes of sudden or partial mania must determine the degree of culpability of the action of the monomaniac, because moral will is subordinate to moral liberty, as this latter is subordinate to the integrity of reason. We sum up our convictions by stating that, supposing that the monomaniac has no interest rationally admissible—no motive which could impel him to an act condemned by sound reason, this act, if it be consummated, ought to be attributed to mania, and not exclusively to impulse, to irresistible propensity, or to depraved will. And we repeat once more, that this doctrine of jurisprudents (monomania without delirium) plainly tends to destroy free will, or the moral liberty of human actions."

It may perhaps be objected, "If perception, judgment, and moral sensibility can be singly perverted or abolished, why cannot the *will*, which is a compound of the intellectual and moral being, why cannot it experience by *itself* the same perturbation, the same annihilation?" Now this is the most specious argument that could be opposed to us. Without hesitation we admit, with all medical-jurisprudents, and, in fact, all medical men, that the will can be more or less weakened, perverted, or depraved; but we maintain that that perversion of the will, *with-*

*out motive*, never proceeds to action, without there being present at the moment of execution some intellectual trouble, or suspension or loss of free-will. We say without motive or interest, for every man who acts without these stimulants does not act like a reasonable being. He is in opposition to the laws of common sense; that is to say, he is what is called mad. Dr. Morin, in his thesis, has given the following anecdote to illustrate monomania without delirium:—

"During the wildest excesses of the French revolution, the rabble, after the massacres of the prisons, forced themselves like madmen into the Bicêtre Asylum, on the pretext of delivering certain victims of the ancient tyranny, confined with the insane. They ran in arms from cell to cell. They spoke to the inmates, and left them if the alienation was evident. But one of the patients, chained to the wall, attracted their attention by his sensible and reasonable language and his bitter complaints. Was it not abominable that he should be kept in irons, and be treated as if he were mad? He defied any one to reproach him with the slightest act of extravagance. It was, he said, the most revolting injustice. He begged the strangers to put a stop to such oppression, and be his liberators. By such management he excited violent murmurs among the armed mob, and cries of vengeance against the superintendent of the hospital. They dragged him to the place to give an account of his conduct, and all swords were directed against his bosom. They accused him of having lent himself to the most cruel excesses, and they stopped his mouth the moment he began to justify himself. It was of no use for him to cite similar examples of insane people not delirious, but very dangerous from their indiscriminating fury. They replied by imprecations; and if it had not been for the devotion of his wife, who covered him, so to speak, with her body, he would have fallen several times beneath their swords. To their order the chains were knocked off, and the released patient was conducted in triumph, amid resounding cries of '*Vive la republique!*' The sight of so many armed men, their noisy and confused jabbering, with their faces suffused with drunken excitement, roused the dormant fury of the maniac. He snatched with a vigorous arm the sword of one who stood near him, dealt his blows right and left, and made the blood flow; and if he had not been overpowered, he would this time at least have revenged outraged humanity. The horde of barbarians took him back to his cell, and seemed to yield with a blush, for once, to the voice of justice and experience."

It is plain, observes our author, that there was here a sudden explosion of maniacal delirium, and a suppression of reason; and if this man had committed homicide, this material murder would have had no character of criminality. Here are other examples, more to the point, cited from various writers.

"A servant threw herself at the feet of her mistress, and asked her permission to be allowed to leave her house. She confessed that every time she undressed the child confided to her care, and for whom she felt all a mother's fondness, she experienced an almost irresistible desire to destroy it.

"A mild, amiable man, of distinguished merit, prostrated himself every day before the altar, to implore Divine goodness to deliver him from an inclination so villanous that he would never tell what it was.

"A countrywoman, lately delivered and suckling her infant, suddenly felt herself seized with a desire to kill it. She held it in her hands and fixed her eyes on it, just ready to yield to the impulse. She trembled with horror, and ran out, in the fear that she should not be able to master her feelings. Returning to give it the breast and again tempted, she fled bewildered once more. During a whole day she struggled against the destructive ideas with which her soul was filled."

In these cases the morbid impulses have been suitably resisted, and have not, therefore, been followed by the final act. This is what should always be done, and would be successful by the help of religion.

"Some years back," says M. Debreyne, "I was consulted by a man in opulent circumstances, who described himself as perfectly happy and exempt from all source of trouble and annoyance, with the exception of a single subject which tormented him. This was a desire, a propensity, a violent temptation, to cut his throat every time that he shaved himself. He imagined that if he did this deplorable and silly act, he should experience an *indescribable pleasure.* Often was he obliged to throw the razor from him. This is, in fact, the best way to act, when there is not sufficient moral or intellectual power either to subdue or out-reason such depraved instincts and desires."

The following case in point is furnished by M. Esquirol :—

"A little girl, eight years of age, manifested the resolution to destroy her mother-in-law. When taken to M. Esquirol, this eminent physician put a series of questions to her, to which she replied artlessly, with the simplicity and calmness of childhood. She declared that she bore no hatred to the wife of her father, that she was most grateful for her kindness, but that whenever she saw her she wished to kill her. The mere sight of that woman was enough to excite in her the horrible idea. M. Esquirol, after a skilful cross-questioning with tact which is beyond imitation, managed to get out of her the obscure and almost forgotten origin of this fearful monomania. He learned that some expressions of anger and hatred, most probably accompanied with violent gesticulation, had been used, *six years before*, by the relations of her father, against the person whom he was going to take for his second wife. The child was then only two years of age, and this violent scene took place in her presence. An impression was produced. A nervous excitement, corresponding with that impression, was induced each time that her mother-in-law came in her sight. An abnormal vicious association was established between the affective impression experienced at the age of two years, and the sensorial impression determined every day by the presence of her mother-in-law. Hence the reproduction of the initiative innervation for which the child was taken to M. Esquirol. Without malice, irreproachable in conscience, pure in thought and will, she was moved by a blind automatonism. She obeyed an impression or impulse, at once obscure and powerful, of which murder was like to have been the result. What subject for serious meditation touching the pathogeny of that form of nervous surexcitation! What a case for grave reflection on the impressionability of man during his most tender infancy!"

The necessity of being most careful in our conversation and manners before young children, must be evident from this. It was Aristotle, we believe, who originally inculcated the advantages of commencing education in the cradle. The lessons for good or evil taught in early infancy are never forgotten. The first signs marked on the unsullied tablets of the mind are seized with eagerness and stored with care, as first love is said to take the quickest and the deepest hold upon the maiden heart.

"The reader will perceive," says M. Debreyne, "from this explanation, that our opinion differs from that of the physicians who acknowledge two kinds of homicidal and suicidal monomania; one accompanied with delirium, the other without delirium. For ourselves, we reject that without delirium during the act. On the other hand it may here be mentioned, that the greater number of magistrates and jurisprudents reject altogether the doctrine of monomania. As, for instance, the advocate-general in the case of Henrietta Cornier, and M. Dupin, in the affair of Darzac, where they stated that 'monomania is a chimera, a mere phantom, raised up at one time to snatch the guilty from the just severity of the law, at another to arbitrarily deprive a citizen of his liberty.' This negative, absolute, exclusive opinion is still not correct. It is confuted by hundreds of facts which incontestably prove the existence of homicidal and suicidal monomania, or if you prefer the expression, that impulse more or less powerful and imperious to commit criminal acts always co-existent with free will or moral liberty, which is not destroyed except at the very moment of the act. We think that it would be better to say, with M. Collard de Martigny, that 'monomania is a passion which can be stifled only during its infancy.'"

Let us, then, adopt a middle course, an opinion midway between that of the doctors and that of the lawyers. Let us say that homicidal and suicidal monomania really exists, but never without delirium; at least, when it realizes its object and proceeds to action. It is then only that the impulse, no longer to be repressed or controlled, can be said to be *irresistible.* M. Esquirol himself, who is no slight authority in this matter, wrote, in the year 1821, these remarkable words: "It is asserted that there are individuals who desire to commit suicide by a sort of irresistible impulse. I have never seen such individuals. I dare believe that if those individuals had been better studied who were said to have obeyed an insurmountable propensity, the motives for their determination would have been discovered." But such a case occurred in 1839. In that year the *Gazette des Tribunaux* and other journals revealed to France a fact both extraordinary and important. This was it. A young girl was condemned, in 1826, to hard labour for life, for having, in cold blood, cut off the head of a neighbour's child. "In the absence," says the *Gazette des Tribunaux,* "of all conceivable motive for the commission of such a crime, the faculty declared that the reason of the accused presented unequivocal signs of mental alienation."

Since this condemnation, many writers on legal medicine have placed this in the list of cases of homicidal monomania. In more than one criminal trial this bloody record has been adduced for the defence of certain accused persons; but the whole truth was disclosed after a while, to the discomfiture of the new theory of monomania.

It appeared, according to the same journals, that the girl confessed, that after having been deserted by her lover for another woman whom he married soon afterwards, she conceived the idea and matured the project of a horrible vengeance: that she consummated her crime in murdering the child of her rival and former sweetheart. She was, she said, a little touched by the cries of the poor infant; but she persisted in her vengeance. This case, and the conclusions drawn from it, together with the avowal of the culprit, are calculated to give rise to grave and serious reflection in the minds of both advocates and psychologists.

